# Microfluidic technologies for cell deformability cytometry

**DOI:** 10.1002/SMMD.20220001

**Published:** 2022-12-22

**Authors:** Hanxu Chen, Jiahui Guo, Feika Bian, Yuanjin Zhao

**Affiliations:** ^1^ Department of Clinical Laboratory Nanjing Drum Tower Hospital School of Biological Science and Medical Engineering Southeast University Nanjing, Jiangsu China; ^2^ Oujiang Laboratory (Zhejiang Lab for Regenerative Medicine, Vision and Brain Health) Wenzhou Institute University of Chinese Academy of Sciences Wenzhou Zhejiang China

**Keywords:** biomechanical, deformability cytometry, high‐throughput, microfluidic, single‐cell

## Abstract

Microfluidic detection methods for cell deformability cytometry have been regarded as powerful tools for single‐cell analysis of cellular mechanical phenotypes, thus having been widely applied in the fields of cell preparation, separation, clinical diagnostics and so on. Featured with traits like easy operations, low cost and high throughput, such methods have shown great potentials on investigating physiological state and pathological changes during cellular deformation. Herein, a review on the advancements of microfluidic‐based cell deformation cytometry is presented. We discuss several representative microfluidic‐based cell deformability cytometry methods with their frontiers in practical applications. Finally, we analyze the current status and propose the remaining challenges with future perspectives and development directions.

## Graphical abstract

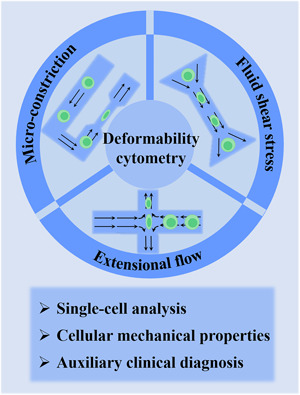

Microfluidic detection methods for cell deformability cytometry have been regarded as powerful tools for single‐cell analysis of cellular mechanical phenotypes, thus having been widely applied in the fields of cell preparation, separation, clinical diagnostics and so on. Featured with traits like easy operations, low cost, and high throughput, such methods have shown great potentials on investigating the physiological state and pathological changes during cellular deformation. Herein, a review on the advancements of microfluidic‐based cell deformation cytometry is presented. We discuss several representative microfluidic‐based cell deformability cytometry methods with their frontiers in practical applications. Finally, we analyze the current status and propose the remaining challenges with future perspectives and development directions.

1

**Key points**

- We presented novel microfluidic technologies for cell deformability cytometry.
- Advantages of several microfluidic‐based cell cytometry methods were reviewed.
- Current status and remaining challenges of cell cytometry were proposed.

## 2 INTRODUCTION

**FIGURE 1:**
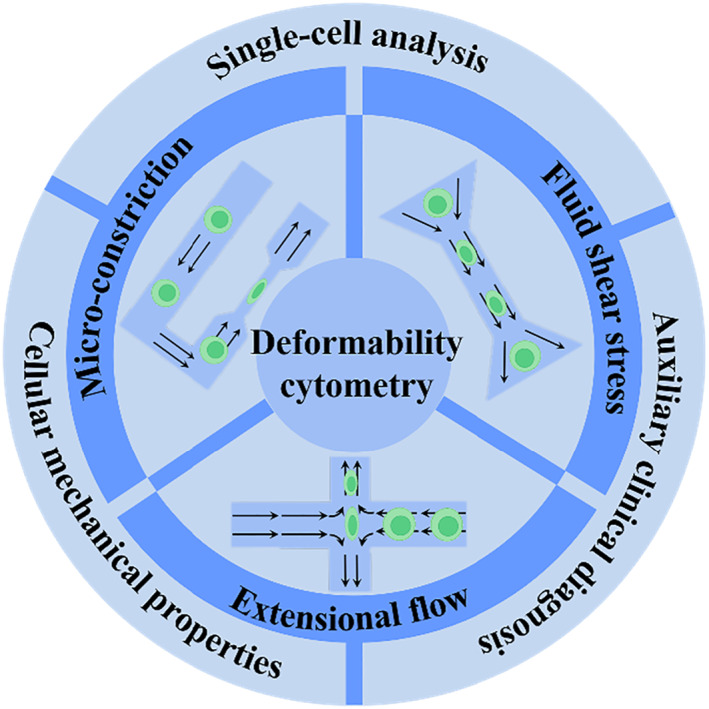
Scheme of the microfluidic‐based deformability cytometry methods and biomedical applications.

Microfluidic detection methods for cell deformability cytometry have been considered as powerful tools for analysis on cellular mechanical properties at single‐cell level. Such mechanical observations directly reflect the cellular growth status and certain critical functions, which could be further associated with pathology at the cellular level. Thus these methods have been widely applied in a variety of fields, including basic research, cell preparation, clinical diagnostics and so on.^1^, ^2^, ^3^, ^4^ Compared with traditional technologies like atomic force microscopy (AFM), micropipette aspiration, microbead rheometry, and optical tweezers/traps, microfluidic‐based deformability cytometry methods have shown advantages like easy operations, low cost and high throughput, thus becoming a more suitable tool for investigation of the cellular deformation along with physiological state and pathological changes (Figure 1).^5^, ^6^, ^7^, ^8^, ^9^ Specially, last decades have witnessed the burst of the advancements of such microfluidic‐based technologies, including constriction deformability cytometry, fluid shear deformability cytometry, and extensional flow deformability cytometry.^10^ In addition, to meet practical application requirements, high‐speed time‐resolved imaging and computer‐assisted automated processing procedures are also developed to undertake efficient and accurate analysis of mechanical deformation.

Evolved with a distinctive trait of autonomous measurements, microfluidic‐based deformability cytometry can achieve breakthroughs on the limited throughput, thus exhibiting great potentials especially in the field of auxiliary clinical diagnosis. Herein, a review on the advancements of microfluidic‐based cell deformation cytometry is presented, covering several main adopted methods with their frontiers in practical applications. Finally, we analyze the current status and propose the remaining challenges with future perspectives and development directions on the basis of the achievements.

## 3 CONSTRICTION DEFORMABILITY CYTOMETRY

**FIGURE 2:**
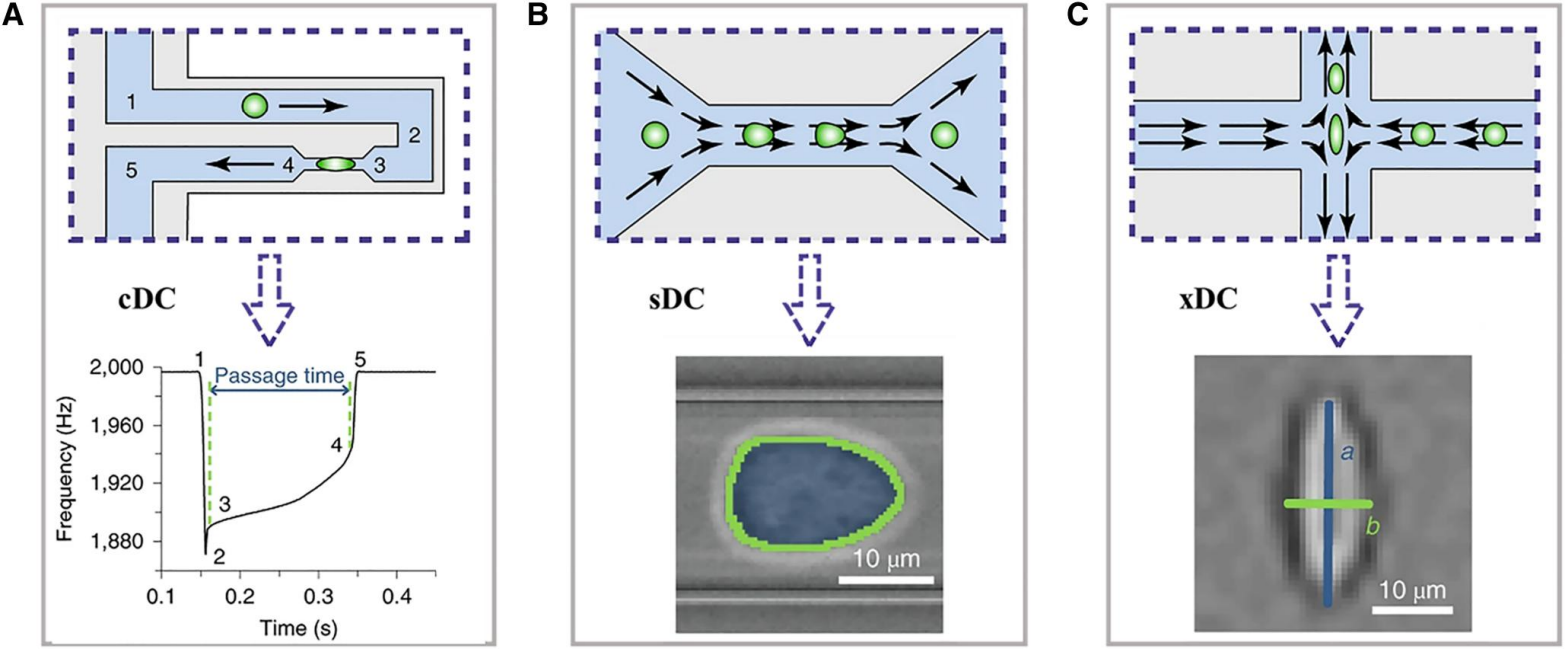
The schematic operation principles of three types of microfluidic technologies for cell deformability cytometry, including constriction deformability cytometry (A), fluid shear deformability cytometry (B), and extensional flow deformability cytometry (C). The bottom rows represent the typical signals of each method. Reproduced under terms of the CC‐BY license.^10^ Copyright 2020, The Authors, published by Springer Nature.

Typical microfluidic‐based constriction deformability cytometry (cDC) method relies on driving target cells in a flow field through a narrow constriction bearing a smaller size than the cell diameter, as shown in Figure 2A. Due to the constraint of the confined wall with specific geometry, cells would undergo respective mechanical deformability based on the surface friction, stiffness, viscoelasticity, and adhesive property. By means of recording parameters like transit time, cellular shape size, entry/transit velocity, and relaxation time (cell restoring force), the cell deformability could be directly calculated or deduced.^11^, ^12^, ^13^, ^14^, ^15^ Generally, cDCs are always coupled with following main external detection approaches involving optical imaging, electrical resistance sensors (impedance or conductance), and suspended microchannel resonant (SMR). Furthermore, finite‐element modeling could be integrated to improve the measurement accuracy of mechanical properties. Hence, such microfluidic‐based cDC methodology has been widely used for the assessment of cell lines, including blood cells (suspended erythrocytes, leukocytes, and neutrophils), invasive/noninvasive cancer cells, and so on.

**FIGURE 3:**
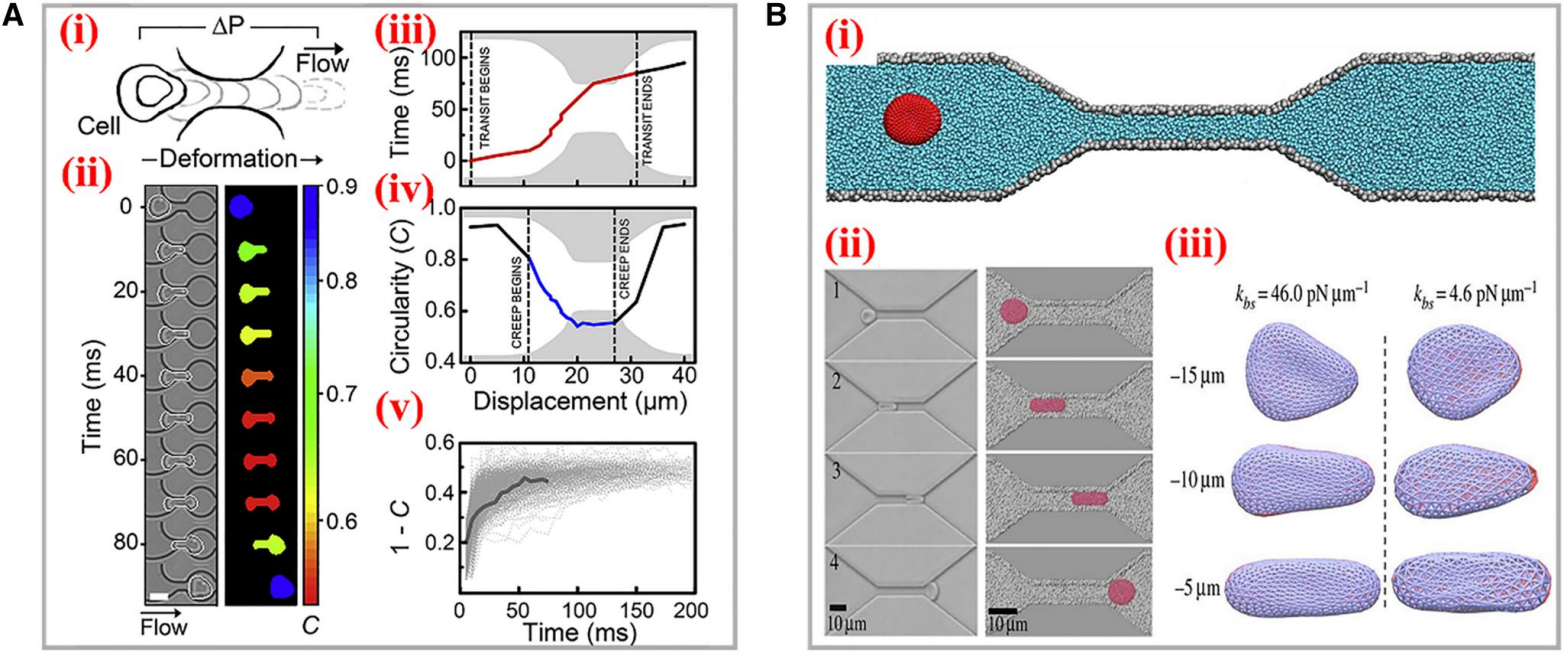
Optical imaging of the cell deformation process. (A) Scheme (i) and time sequence (ii) of a single cell passaging through the micron‐constriction with a pressure‐driven flow. (iii‐v) The characteristic parameters achieved during deformation. Reproduced with permission.^16^ Copyright 2017, Biophysical Society. (B) (i) Schematic representation of a red blood cell traversing across the microchannel. (ii) The experimental images (left) and simulation (right) data of the cellular shape during the traversing process. (iii) Bilayer–cytoskeletal detachment for the cell at different elastic interactions. Reproduced with permission.^18^ Copyright 2014, The Authors, published by the Royal Society.

Among these methods, optical imaging *via* high‐speed charge coupled device (CCD) for capturing image sequences has shown great advantages like easy operations, direct analysis, and high throughput. For example, Rowat and his colleagues realized the quantitative measurement of cell mechanotype by recording high frame rate time sequence of human promyelocytic leukemia (HL‐60) cells passaging through micron‐constriction, as presented in Figure 3A.^16^ The cell position and shape were tracked by color thresholding and further extracted with morphology. It was worth mentioning that the externally applied stress was calibrated by gel particles with standardization elastic moduli, and thus the deformation response of the cell could be precisely determined. In addition, several drugs for cytoskeleton perturbing (cytochalasin D, jasplakinolide and blebbistatin) were incorporated to investigate the corresponding mechanical deformability, which could serve as a biomarker within disease biological process. Astonishingly, Walter’s group achieved a direct quantificational sensory of cell deformation by introducing an elastic microflap cantilever as the force sensor inside the boundary of constrictions.^17^ Cells were flowed into microrestriction and exerted force on the cantilever. Both the deformation of cells and cantilevers were optically tracked for exact mechanical testing. Intriguingly, dynamic simulation modeling before optical experiments could better predict the deformable motions of cells and optimize the parameters of constrictions. Based on the dissipative particle dynamics simulation technique, Karniadakis et al. established a red blood cell model containing two components (lipid bilayer and cytoskeleton) to reproduce the squeezing deformations through narrow capillaries and study the biomechanics and rheology (Figure 3A).^18^ The dynamic simulations were performed, and the shape deformation under different bilayer–cytoskeletal elastic interaction coefficients were in accordance with optical imaging. This model was demonstrated with the capacity to mimic the mechanical properties and deformation behaviors of red blood cells in hematological diseases.

**FIGURE 4:**
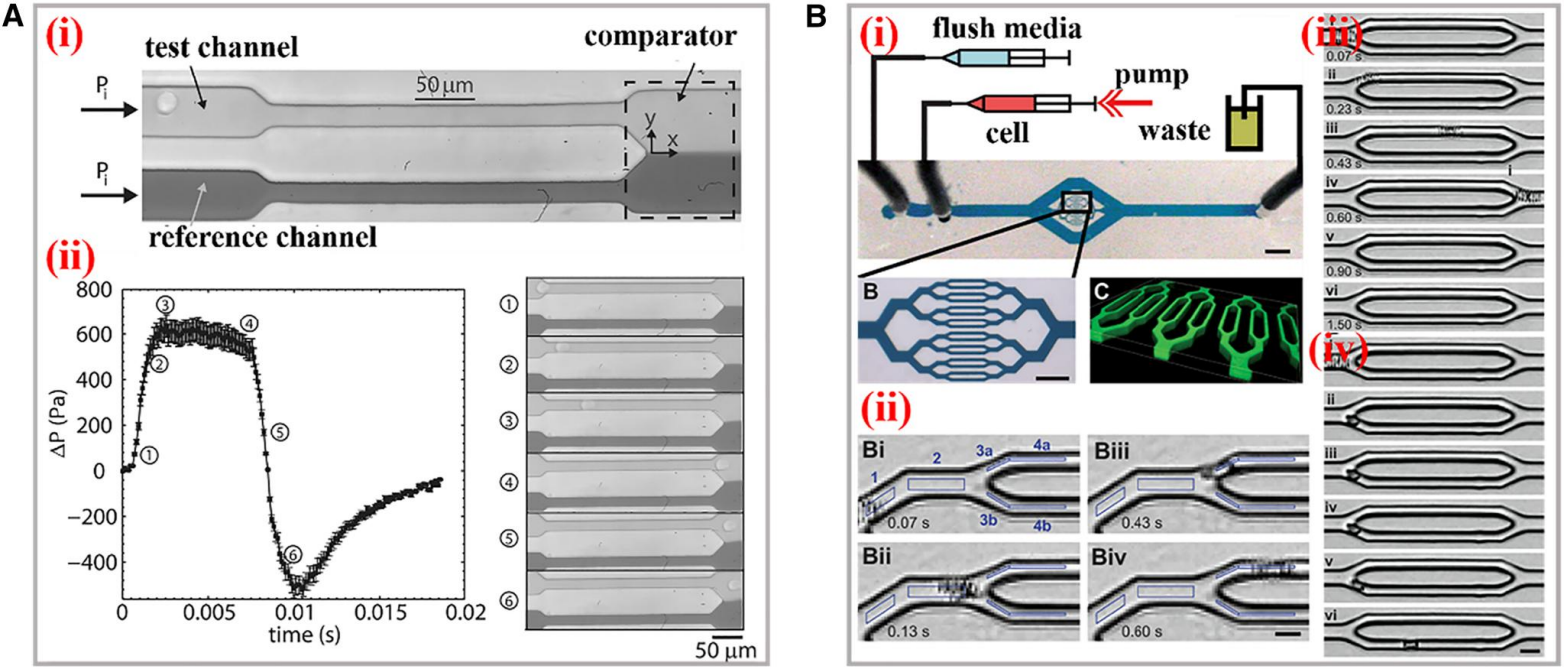
Practical applications of cDCs. (A) (i) The structure and working mechanism of the microfluidic cell squeezer. A balanced interface in the comparator region between the fluids in the reference and test channels. (ii) The curve between excess pressure drop and time. The optical images of different states of the interface within the squeezer corresponding to the cellular deformations. Reproduced with permission.^21^ Copyright 2013, AIP Publishing. (B) (i) The scheme of the biophysical flow cytometer device. (ii) This visual tracking of neutrophil. The images of neutrophil transiting through microchannels before (iii) and after (iv) exposure to the inflammatory mediator increases. Reproduced with permission.^24^ Copyright 2008, The Royal Society of Chemistry.

Certainly, apart from constrictions with fixed geometry, there existed microfluidic platforms bearing flexible constrictions for wider applications, involving analysis of diverse cell lines with different morphologies and tunable size‐independent deformation on a single cell. For instance, Guan’s group designed a microfluidic device encompassing adjustable constrictions for deformability detection of cells possessing large size variations.^19^ The height of constrictions could be controlled by a pressure regulator in real time. Such a device was applied to observe the deformation of two similar human breast cancer cell lines (MCF‐7 and MCF‐10A) that differed in structures and sizes, and the stiffness profiling results enabled to distinguish diverse cell populations. Similarly, Wang and his teamworkers utilized a pneumatically driven membrane‐based active valve to obtain cellular electrical properties (specific membrane capacitance and cytoplasm conductivity).^20^ The membrane deformed by negative pneumatic pressures to change the height of the constriction channel, which also prevented the clogging of cell aggregates at the entrance. The variations of electrical properties caused by deformation within constrictions at single‐cell level were successfully investigated. Instead of directly observing deformed cells, probing the distribution of fluid pressures around two sides of constrictions has been considered as another effective detection manner. For example, Vanapalli’s group constructed a microfluidic cell squeezer device to measure the pressure drop caused by the traverse of brain cells through confined constriction, as presented in Figure 4A.^21^ Two completely similar parallel fluidic channels bearing constriction regions were connected at the end, and one served as a testing channel and the other acted as a reference channel. When cells entered the constrictions of the testing channel, the pressure drop would be generated, and thus the initial balanced liquid interface at the end would appear as a corresponding displacement. By optically measuring the numerical value of such an excursion, the pressure drop was evaluated and the cell deformation could ulteriorly be characterized.

Especially, it is necessary to process effective single‐cell deformability cytometry among thousands to millions of cells within short periods. The target cells might account for only a small minority of the analyte samples. Hence, sufficient throughput is critical to acquire statistically accurate results. Meanwhile, the processing time should be short as far as possible to avoid unnecessary physical changes of cells in suspensions prior to measurement. If the processing time was not short enough and the analyte cell still stopped in the detection region, the subsequent cells would dash against the front cell under the circumstance of high throughput in the monodirectional narrow constriction. During this process, the analyte cell would generate extra deformation and physical changes, such interference would finally affect the accuracy. One solution to address this problem is to amplify the amounts of parallel detection constriction channels within one platform on the basis of the microfluidic technology that is able to uniformly and precisely control the collateral fluids.^22^, ^23^ Fletcher et al. reported a microfluidic biophysical flow cytometer device for analysis of cell mechanics through constrictions that were geometrically similar to microvascular networks (Figure 4B).^24^ The chip was manufactured with 16 parallel micron‐constriction channels and transit time of individual cells through channels were high‐throughput quantified. It was demonstrated that this system could provide sufficient cell deformation data for clinical applications *via* automated optical imaging analysis on multiplex channels. Analogously, Fabry and his colleagues constructed micron‐constriction arrays and measured the flow speed, cell deformation, and transit time with efficiency of hundred cells per minute.^25^ The constriction area was featured with 8 parallel narrow channels (5 μm in width and 9 μm in length). With the variation of driving pressure and cell size, the transit time represented a power–law relationship, and thus the elasticity and fluidity of cells could be evaluated. Additionally, the mechanical behaviors influenced by external drugs were explored according to above power–law rheology. Theoretically, the throughput of read‐outs would be more efficient with more parallel micron‐constrictions involved. However, due to the restrictions of the view size of the microscope field, the amount of channels cannot be increased infinitely, which brought obstacles for wider applications of optical imaging‐based cDCs.

Alternatively, placing electrical resistance sensors around the constriction region could monitor the electrical signals generated owing to the deformation of cells passaging the constriction.^26^, ^27^, ^28^, ^29^ For instance, Jensen et al. proposed a microfluidic concept where the cells were tethered through a funnel‐shaped constriction.^30^ Two electrodes were fixed on either side of constriction and the electrical resistance was instantaneously recorded with the position. When the cell entered the channel and assumed deformation, the resistance was expected to increase to a peak and returned to baseline after the cell left the constriction. The transmit time could be transferred as the width of signal peak, and the cell diameter and stiffness could be deduced. Sohn and his colleagues developed a microfluidic multiparametric cDC method to monitor the mechanical phenotypes of cancer cells based on four‐terminal current detection. They quantificationally measured several indexes simultaneously involving cell size, resistance to compressive deformation, transverse deformation under strain and relaxation time. Totally four electrodes were placed, where two electrodes served to generate constant voltage and another two monitored the current variations. The characteristic current pulses of different stages were detected and used to estimate the above desired indexes. Moreover, they established a new parameter to optimize whole‐cell deformability on the basis of resistance to compressive deformation, which could be applied for discrimination of malignant/nonmalignant cancer cell lines. In addition, getting rid of the limited field of view, electrode‐based measurement could be easily integrated at a large scale and could further improve the efficiency. Guan’s group integrated a microfluidic cDC sensor with electrode arrays to differentiate health and malaria‐infected red blood cells based on the difference of deformability.^31^ When the individual cell passed through the constriction channel, the ionic current would undergo a respective specific waveform under the external constant voltage. Because of the higher stiffness and less deformation of malaria‐infected red blood cells, the width of the crest would be wider than that of healthy red blood cells. Besides, the evolving progress of malaria‐infected red blood cells could be distinguished according to such a unique waveform. The analysis efficiency was increased with a throughput of 500 cells per second by integrating parallel detection units within one chip.

To improve the detection accuracy, SMR‐based cDCs have been developed and featured with extremely high accuracy compared with above two detection methods. When the cells pass microchannels cantilever, the resonant frequency of SMR would change due to the buoyant mass variation of the single cell. Meanwhile, the position along the channel could be detected with high spatial resolution. By tracking the frequency variation, the cell mass could be precisely calculated. For example, Manalis et al. designed an SMR‐integrated device for single‐cell detection of buoyant mass, passage time (entry and transit), and velocity.^32^ The cell was deformed through the micron‐constrictions that were located at the apex of SMR, and the relationship between frequency and traveling time was recorded where the velocity could be deduced. Furthermore, the power–law curve between buoyant mass and passage time was obtained to effectively identify and differentiate cancer cell lines.

In conclusion, owing to the physical contact between cells and smaller narrow constrictions, the friction, deformability, and retention of cells could be better investigated and observed *via* cDCs. The critical parameter is the geometry size of constrictions which should well match the diameters of cells. Hence, the cDC‐suitable target cells must bear excellent dimensional homogeneity to avoid interference caused by intermittent clogging of channels or loss of signals. To overcome this problem, prior purification and size homogenization would endow cDCs with wider applications for single‐cell analysis.

## 4 FLUID SHEAR DEFORMABILITY CYTOMETRY

Compared with the physical extrusion manner adopted in cDCs, fluid shear deformability cytometry (sDC) is a contactless flow cytometry method, which performs hydrodynamic shear deformation on cells induced by the parabolic velocity distribution and pressure gradient at a cross section of the microfluidic channel.^33^, ^34^, ^35^, ^36^ Typically, the suspension cells are advected by shear flow at constant speed into a channel whose size is slightly bigger than the cell diameter, as shown in Figure 2B. Due to the intrinsic stiffness, the cells would generate mechanical deformation against strong shear stress gradients. Meanwhile, the deformation could cause lift force to drive cells rotating with lateral migration within a continuous flow.^37^, ^38^ Especially, the medium liquid employed in sDC tends to be more viscous in order to generate low Reynolds number below 0.1, where the deformable cells would remain relatively stationary. If the Reynolds number is too high, the flow field would generate turbulent flow, which would cause extra stress on cells and cause unnecessary deformation and lower accuracy. In addition, with the help of high‐speed CCDs, the images of cells with different deformation stages could be obtained in real‐time, and thus sDC methods are also named as real‐time DCs (RT‐DCs). Through subsequent image processing, the quantified cell deformation could be expressed as the following equal:

$$d=1-\frac{2\sqrt{\pi A}}{P}$$

where *A* means the cell projection area, and *P* refers to the cell perimeter. Obviously, the d value is equal to 0 when the cell remains in a spherical state, and when the cell deforms, the d value would go beyond 0.

**FIGURE 5:**
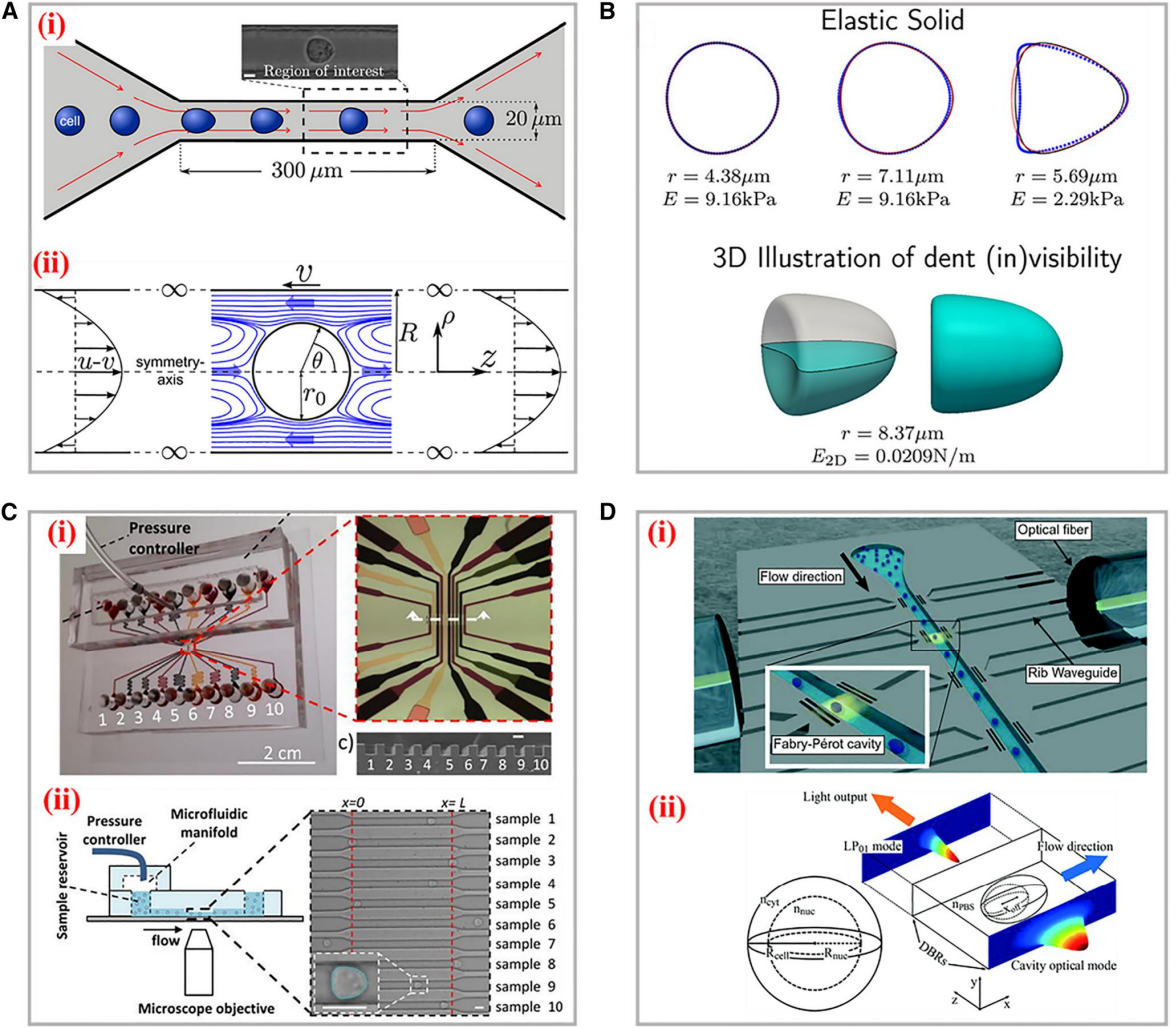
Simulation models and applications of the sDC. (A) The setup of the channel geometry for sDC measurements (i) and the flow field around an advected sphere (ii). Reproduced with permission.^39^ Copyright 2015, The Authors, published by Elsevier Inc. (B) The cellular shapes for the elastic solid model and 3D illustration of the dent at the cell. Reproduced with permission.^40^ Copyright 2017, American Chemical Society. (C) The microfluidic device for multi‐sample deformability cytometry. (i) Optical images of the device embedded with microchannels, sample reservoirs, pressure controller, and microfluidic manifold. (ii) Scheme of the experimental setup. The right panel is the bright field image of cells traveling through the channels. Reproduced under terms of the CC‐BY license.^45^ Copyright 2018, The Authors, published by AIP Publishing. (D) (i) The 3D illustration of the silicon‐on‐insulator RIC device. (ii) Numerical simulation of the cell interaction with optical modes and the reference spherical cell. Reproduced with permission.^52^ Copyright 2019, The Royal Society of Chemistry.

Numerical simulation has been considered as an efficient tool to reproduce the distribution of flow field and precisely predict fluidic parameters within microfluidic channels. For instance, Fischer‐Friedrich and Aland et al. utilized the finite‐element simulation method to explore the geometry shape of cells through microchannels of sDC and extract respective mechanical properties (Figure 5A,B).^39^, ^40^ On the basis of Navier–Stokes equations, they established a numerical model to explain the influences on deformation induced by cell autologous sizes. The cells were described as neo‐Hookean hyperelasticity models where viscoelastic material were wrapped with thin cortex possessing bending stiffness and surface tension. It was found that the cells were inclined to deform toward bullet shape with both small and big sizes. To better understand the hydrodynamic behaviors within sDCs, Guck and his colleagues analyzed shear stress and pressure on the cell surface inside the microchannel.^41^ Two parameters involving size and deformation were efficiently decoupled. Furthermore, the additional definition of isoelasticity lines also contributed to the extraction of desired material properties. Schuster’s group utilized a two‐dimensional symmetric model to investigate the mechanical properties of cells passing through a channel, including elasticity, viscosity, size, and velocity.^42^ Interestingly, the viscosity of the flowing medium could be varied during simulation from constant value to non‐Newtonian fluids, which also served as a critical influence factor on cell deformation. It was worth mentioning that the cell relaxation was successfully characterized to be further mapped as a parameter to discriminate different cells.

As we have described above, integration with imaging snapshot, sDCs could realize real‐time and high throughput mechanical phenotype analysis. For example, Oliver and his coworkers dynamically tracked single cells in the sDC channel with consecutive frames up to 98.^43^ By analyzing the image sequence of the cell shape, they constructed a cellular shape descriptor for the interpretation of cell biomechanical properties. It was demonstrated that the steady state of cell deformation could be determined by cell image traces when the stress relaxation was just dependent on amplitude and duration. Different from traditional narrow straight microchannels applied for sDCs, McGloin et al. proposed a single hydrodynamic microfluidic device embedded multiple indentation channels for the measurement of cell deformability.^44^ Based on the specific geometry of the channels, the inner fluid dynamic was disturbed especially between the opposite indentations, leading to oscillatory stress inducing cell deformation. Besides, the cells would experience periodic deformations when they flow along the channel, which serves as repetitive assessment for highly heterogeneous samples. Certainly, similar as the above cDCs, to enable higher throughput of sDCs, the introduction of multiple parallel channels is still the best choice. Vanapalli’s group reported a novel sDC method encompassing ten sample pathways for simultaneous deformation analysis of multi‐samples, as depicted in Figure 5C.^45^ Such sDC microdevices consisted of sample reservoirs, pressure control modules, and data acquisition systems. The results indicated that different cancer cell lines bear respective deformability with unique metastatic potentials. The analysis efficiency proved to be 100 cells per second, and subsequent data processing consumed 10 min per sample.

Except for the infliction of shear stress on cells to generate deformation, some external supporting stimuli could also be integrated to induce the deformation of cells in suspension.^46^, ^47^, ^48^, ^49^, ^50^ For instance, Marr and his colleagues designed a fluorescence‐activated cell sorters (FACS)‐like detection approach for cellular viscoelastic cytometry.^51^ The red blood cells were flowed into the microchannel, where a linear diode‐bar‐based optical stretcher was applied to perform antipodal sinusoidal deformation forces. The time‐dependent cell strain was statistically measured with a high frequency, which successfully illustrated the increased sampling rate of approximately 1000 cells per hour. Intriguingly, to overcome the restrictions from the defined regions for optical imaging, Leblanc‐Hotte et al. developed a promising sDC discrimination platform for targeting refractive index of cellular deformation (Figure 5D).^52^ The distributed Bragg reflectors containing Fabry–Pérot cavity were able to acquire resonances in the near infrared. For signal propagation, rib waveguides were embedded along the flow microchannel. Upon cells passing through these cavities, the resonance peak would shift toward longer wavelength because of the larger refractive difference between cells and the surrounding fluids. Such a system was demonstrated with a capacity to probe cellular refractive index, effective volume, and deformability with throughput of 5000 cells per second.

## 5 EXTENSIONAL FLOW DEFORMABILITY CYTOMETRY

**FIGURE 6:**
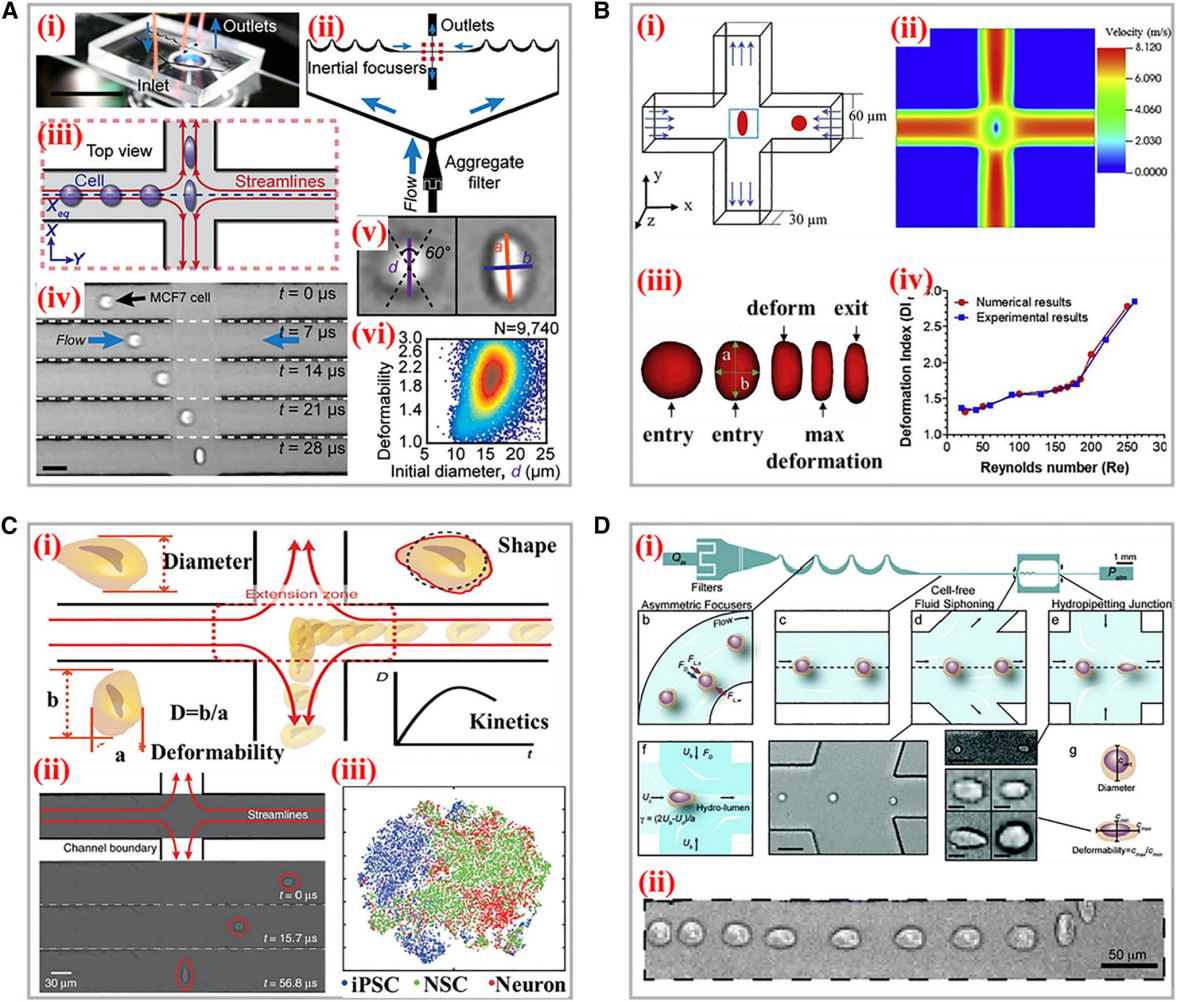
Typical working principle and applications of xDC. (A) (i) The photograph of the microscope‐mounted and fluid‐coupled microfluidic deformability cytometry device. (ii) The structures of microchannels focusing cells before delivering them to the stretching extensional flow. (iii) The scheme of the cellular deformation within an extensional flow previously aligned at an inertial focusing position. (iv) Microscopic images of a single cell entering the extensional region. (v) Definitions of the shape parameters extracted from images. (vi) Density scatter plot of deformability measurements of single human embryonic stem cells. Reproduced under terms of the CC‐BY license.^57^ Copyright 2012, The Authors, published by National Academy of Sciences. (B) (i) Scheme of the cross‐flow channel geometry and cellular deformation under the velocity profiles. (ii) The velocity distribution within the extensional region without the cell present. (iii) The numerical simulation of the cellular shape changes through the cross‐flow channel. (iv) Comparison of the experimental and numerical deformation indexes of cells. Reproduced with permission.^58^ Copyright 2020, Biophysical Society. (C) The schematic diagram of the microfluidic device for analysis of four parameters during cellular deformation process. (ii) High‐speed photography of the deformation. (iii) Visualization of physical phenotypic spaces occupied by iPSCs, NSCs, and neurons. Reproduced under terms of the CC‐BY license.^59^ Copyright 2017, The Authors, published by Springer Nature. (D) (i) The scheme of the hydropipetting method possessing specific microchannel configurations for three working patterns. (ii) Overlaid images of a single cellular deformation including relaxing, and then deforming again in the extensional flow. Reproduced with permission.^60^ Copyright 2013, The Royal Society of Chemistry.

On the basis of sDCs, a similar novel technology named extensional flow deformability cytometry (xDC) has attracted increasing scientific attentions in recent years.^53^, ^54^, ^55^, ^56^ Initially, the cells are previously centered *via* inertial or viscoelastic focusing prior to stretching to ensure the homogeneity delivery of cells into the stress field. In replacement of the traditional long straight microchannels, the xDCs adapt extensional microchannels bearing cross‐shaped structures, where two same flowing mediums are injected from the opposite symmetrical directions and the hydrodynamic stretching is formed at an intersection, as shown in Figure 2C. Such extensional flow field would deform the cells along two axial directions with higher strain levels. Additionally, in contrast to sDC, the selections of flowing medium prefer to be less viscous and the corresponding flowing speeds are relatively much faster, and thus the detection throughput of xDC is extremely efficient. For example, Di Carlo and his coworkers established an automated xDC microdevice to probe deformability at a single cell level based on hydrodynamic stretching, as shown in Figure 6A.^57^ Featured with three functional modules, including inertial focusing, hydrodynamic stretching, and automated image processing, this device was capable to carry out tunable mechanical phenotypes with a throughput at approximately 2000 cells per second. The images of the deformation process and diameter parameters were extracted with an automated analysis algorithm. The leukocytes and malignant cells in pleural effusions were efficiently assayed by observing the cells deformability under cancer disease states. To better explain the underlying mechanism of extensional stretching‐induced deformation, Khismatullin et al. simulated the flow distribution at intersection position and optimized several fluidic parameters (inlet velocity, shear elasticity, and Reynold number) that influence the deformation index of cells (Figure 6B).^58^ The motion and deformation processes of cells were reproduced in the numerical model and the mechanical properties of MCF‐7 cells were characterized. A new index, namely elongation index, was defined based on the relationships between the cell diameter, shear elasticity, and offset distance. In addition, this index was demonstrated to be an effective parameter to describe the cell deformability in a randomized study. Di Carlo’s group proposed a noble multi‐parametric xDC technology and successfully investigated over 21 different cell motion and morphology‐derived parameters to describe the mechanical phenotypes of several types of cells, as presented in Figure 6C.^59^ Four critical indexes involving average diameter, morphology, deformability, and kinetics were extracted from these parameters by high‐speed videos. Furthermore, the high‐dimensional physical phenotypic spaces were visually mapped with interactive stochastic neighbor embedded to discriminate different progeny and the pathways.

Certainly, the geometry structures of channels within xDCs could be flexibly adjusted. Dudani and his teammates designed a perpendicular high‐speed pinched‐flow‐based xDC method to perform hydrodynamic stretching (Figure 6D).^60^ The cells were previously inertially focused and then squeezed into the hydro‐pipetting region. It was worth mentioning that the integration of hydraulic circuit enabled tunable stretching forces, which were induced by self‐sheathing flow from a single fluid input within the same microfluidic channel. Particularly, this system was endowed with the ability to switch stretching patterns involving extensional and pinched‐flow with a high throughput reaching 65 thousand cells per second. However, the extremely high efficiency of measurement demands CCDs with better performances to capture the single cell undertaking high‐velocity motion and deformation. Meanwhile, subsequent processing of a huge amount of acquisition image data also brings great challenges for future development of xDCs, where new image‐analysis implementations are expected to eliminate this bottleneck.

## 6 CONCLUSION AND PERSPECTIVE

Microfluidic‐based deformability cytometry methods represent a new paradigm for the investigation of cellular biomechanical properties and clinical diagnosis applications owing to the merits like excellent accuracy, sensitivity, and high throughput. For example, the cellular stiffness, which tends to differ under a normal and pathological state, could be directly observed and measured under deformation situations *via* above reviewed microfluidic technologies. Such an index could be furthermore adopted as auxiliary criteria for precise medical diagnosis. Although numerous microfluidic deformability cytometry technologies have been developed for analysis of mechanical phenotypes at the single‐cell level, a huge gap still remains between laboratory experiments and practical applications. For instance, the narrow micro‐constrictions widely utilized within cDC methods are primarily applied for cell types bearing uniform size, which means the channels are otherwise easy to be clogged due to inhomogeneous biofluids. Especially, the clinical samples tend to contain complex cellular components, and sometimes interested target cells account for a relatively tiny fraction. Due to the obstacles to purify desired cells from original samples, clinical detection of some early diseases through microfluidic‐based deformability cytometry methods still faces great challenges. Moreover, the necessary pretreatment of suspensions containing cells should be carefully operated to satisfy the demands of a uniform driving force. As to sDC and xDC methods, the cell deformation under hydrodynamic stretching are sensitive to the fluid stress field. Hence, the precise control on the particular flow field plays the critical role of influencing cellular deformation, including the geometrical constructions, inlet velocity, flowing viscoelasticity, and so on. In addition, although plenty of parameters during deformation could be real‐time acquired, the main deformability properties should be decoupled from the contributions of both the cellular size and inner cytoskeletons. Thus, there is still a long way to go for microfluidic‐based deformability cytometry to be applied in practical clinical applications.

Apart from the manner of performing external forces on deformable cells, the tracking technologies on rapid cellular deformation responsiveness also deserve attention. Typical integration of high‐speed CCDs to capture instantaneous image sequences or videos actually realize the real‐time recording of cellular motions and deformations. However, the restricted microscopy field of view limited the measurement procedures at a certain region. Besides, the detection accuracy of above three methods should be further improved. Tiny cellular deformation could be precisely recognized and analyzed from picture sequences bearing a high resolution. Except for the deformation process, cell behaviors, like the initial cellular motions before deformation, extra drug or virus‐induced deformation, and the following relaxation, are also worthy of study to deepen underlying mechanisms of some disease models. Meanwhile, insight regarding how to efficiently extract desired indexes of deformation from the original images information for further analysis can provide infinite opportunity to meet the requirement for the high throughput of clinical samples. Thus, the relative algorithm, software, and hardware are urgent to be developed to enable precise measurement and visualization characterizations. Last but not least, more efforts should be focused on the investigation of the inner‐cellular bio‐mechanisms on deformation, thus promoting the development of microfluidic‐based cellular deformation cytometry.

## AUTHOR CONTRIBUTIONS

Yuanjin Zhao conceived the idea; Hanxu Chen wrote the manuscript and edited the figure; Hanxu Chen, Jiahui Guo and Feika Bian revised the manuscript.

## CONFLICTS OF INTEREST

There are no conflicts to declare. Yuanjin Zhao is a member of the *Smart Medicine* editorial board.
